# Membrane Fouling Phenomena in Microfluidic Systems: From Technical Challenges to Scientific Opportunities

**DOI:** 10.3390/mi12070820

**Published:** 2021-07-13

**Authors:** Andrea Iginio Cirillo, Giovanna Tomaiuolo, Stefano Guido

**Affiliations:** 1 Dipartimento di Ingegneria Chimica, dei Materiali e della Produzione Industriale, University of Naples Federico, 80125 Naples, Italy; andreaiginio.cirillo@unina.it (A.I.C.); steguido@unina.it (S.G.); 2 CEINGE Advanced Biotechnologies, 80131 Naples, Italy

**Keywords:** micro-scale reactors, microfluidics, fouling, biofouling, membranes, filtration, in situ observation

## Abstract

The almost ubiquitous, though undesired, deposition and accumulation of suspended/dissolved matter on solid surfaces, known as fouling, represents a crucial issue strongly affecting the efficiency and sustainability of micro-scale reactors. Fouling becomes even more detrimental for all the applications that require the use of membrane separation units. As a matter of fact, membrane technology is a key route towards process intensification, having the potential to replace conventional separation procedures, with significant energy savings and reduced environmental impact, in a broad range of applications, from water purification to food and pharmaceutical industries. Despite all the research efforts so far, fouling still represents an unsolved problem. The complex interplay of physical and chemical mechanisms governing its evolution is indeed yet to be fully unraveled and the role played by foulants’ properties or operating conditions is an area of active research where microfluidics can play a fundamental role. The aim of this review is to explore fouling through microfluidic systems, assessing the fundamental interactions involved and how microfluidics enables the comprehension of the mechanisms characterizing the process. The main mathematical models describing the fouling stages will also be reviewed and their limitations discussed. Finally, the principal dynamic investigation techniques in which microfluidics represents a key tool will be discussed, analyzing their employment to study fouling.


**Contents**

1. Introduction ………………………………………………………………………………………………………2 1.1. The Fouling Phenomena ……………………………………………………………………………………3 1.2. Microfluidic Membrane Devices …………………………………………………………………………42. Fouling: Stages and Interactions ………………………………………………………………………………5 2.1. The Pre-Fouling Stage ………………………………………………………………………………………6 2.2. Membrane Adsorption and Pore Blocking ………………………………………………………………8 2.3. The Gel/Cake Layer Formation ……………………………………………………………………………9 2.4. Biofouling ……………………………………………………………………………………………………113. Fouling Mathematical Modeling ………………………………………………………………………………12 3.1. Resistance-in-Series Model …………………………………………………………………………………12 3.2. Blocking Filtration Laws and Cake Filtration Model ……………………………………………………13 3.3. Combined Models …………………………………………………………………………………………154. Dynamic Investigation Techniques ……………………………………………………………………………17 4.1. Direct Microscopic Observation …………………………………………………………………………18  4.1.1. Bright-Field Microscopy ………………………………………………………………………………19  4.1.2. Fluorescence Microscopy ……………………………………………………………………………20  4.1.3. Confocal Laser Scanning Microscopy ………………………………………………………………22 4.2. Optical Coherence Tomography …………………………………………………………………………23 4.3. Nuclear Magnetic Resonance Imaging …………………………………………………………………24 4.4. Other Emerging Techniques ………………………………………………………………………………25  4.4.1. Raman Spectroscopy …………………………………………………………………………………26  4.4.2. Fourier Transform Infrared Spectroscopy …………………………………………………………27  4.4.3. Ellipsometry ……………………………………………………………………………………………27  4.4.4. X-ray Microimaging ……………………………………………………………………………………285. Perspectives ………………………………………………………………………………………………………29References ……………………………………………………………………………………………………………29

## 1. Introduction

Micro-scale reactors (MRs) are attracting increasing attention thanks to the combined advantages of continuous flow operations over traditional batch processing in terms of efficiency [1], product quality [2] and sustainability [3,4], with those typical of microfluidics allowing in situ observations and unprecedented control of transport phenomena, chemical reactions and operating conditions [5]. Being made of channels with the characteristic dimension <1 mm, MRs allow continuous operations with a reduction of reactants, wastes and energy consumption [6], with the high surface to volume ratio ensuring high heat and mass transfer, thus resulting in a higher reaction yield and selectivity with respect to classical batch processes [7]. Further advantages are higher safety [8], faster time-to-market process designs and flexibility [9]. Therefore, MRs can be recognized as the main path towards process intensification [10] in all the fields that manage small capacities, such as fine chemicals and the pharmaceutical and biomedical industries [11].

However, such enthusiasm about MR technology is partially hindered by major issues related to flow confinement, such as solid handling, which asks for a better comprehension of the fluid phenomena at the micro-scale [12]. Indeed, since the beginning of the development of MRs, the issue of solids handling was considered a major challenge. Particulate flow could lead to the undesired deposition and accumulation of particles (foulants) on clean surfaces, a phenomenon known as fouling. Although quite studied in the literature, fouling remains a largely unsolved and challenging problem, and most of the publications are focused on very specific fouling problems, making it difficult to derive general criteria and parameter dependencies [13]. The complex interplay of physical and chemical mechanisms [14] governing the onset and evolution of fouling is indeed still to be fully unraveled and the role played by the properties of foulants, surface material and operating conditions is an area of active research. In MRs, fouling can easily lead to clogging, which causes progressive flowrate reduction, taking a heavy toll in terms of increased energy requirements and operational costs [15].

The problem of fouling becomes even more detrimental for all the applications where porous media, and especially membranes, are present, from petroleum reservoirs and oil wells [16] to cultural heritage preservation [17,18]. As a matter of fact, membrane technology is a key route towards efficient separation operations and process intensification, thanks to the fact that it requires low space and low energy (since phase separation is not needed), and allows to work in continuous flow. In this scenario, membrane-based processes have the potential to replace conventional separation procedures, with significant energy savings and reduced environmental impact, in a broad range of applications, such as water and wastewater treatment, batteries, biotechnology and the pharmaceutical industry [19].

### 1.1. The Fouling Phenomena

Fouling is defined as the accumulation of undesired material at an interface, e.g., the contact region between a fluid and a solid [20]. Material deposition can cause a series of chemical and physical changes at the interface, leading to a loss in heat and mass transfer, as well as to a pressure buildup [21,22]. This is a transient process, beginning as soon as fluid comes into contact with a surface, which eventually becomes so fouled that it completely loses its functionality. Fouling can be caused by a plethora of organic and inorganic species, commonly termed foulants, in the form of particulates, colloids, dissolved components and biological microorganisms [23,24,25,26].

A wide range of industrial and commercial applications relies on the processing of fluids prone to fouling. In heat exchangers, foulant deposition can take place on both the hot and cold sides of the apparatus, compromising the thermo-hydraulic performances of the system [27,28]. In fact, the accumulation of substances such as organic matter contained in process currents (e.g., crude oil), or the debris in cooling water, act as an insulator, increasing the resistance to heat transfer. Moreover, the increasing thickness of the deposits over time leads to a reduction in the cross-sectional area with a consequential rise of the pressure drop. In 1972, Taborek et al. indicated fouling as the major unresolved problem in heat transfer, but its relevance is still very high because of its impact in terms of energy losses, fuel consumption and emissions [29,30]. A similar influence of fouling on thermal and hydraulic resistances also takes place in steam cracking reactors, where the formation of coke on the inner wall of the tubular reactors represents the major reason of process inefficiency [31]. Indeed, due to fouling, systems need to be periodically halted and decoked, procedures that negatively affect not only process economics but also the reactor lifetime [32].

**Figure 1:**
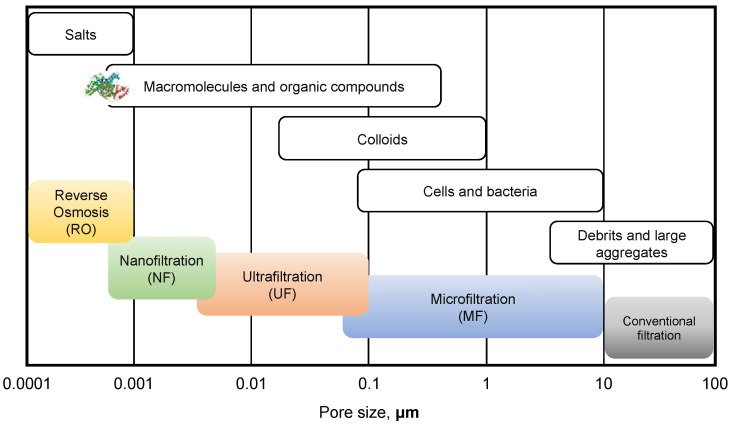
Nominal pore size classification of membrane processes and characteristic dimensions of various common foulants. Despite the categorization, upper and lower size limits usually overlap between two adjacent processes due to pore size distributions. The figure also shows the most suitable process for each foulant category.

However, among all the applications where fouling constitutes a technical challenge to process performances, membrane systems are the ones in which this phenomenon represents the most critical issue and limiting condition. As a matter of fact, membrane processes are based on the ability of a membrane to regulate the permeation rate of species through the pores, so that the accumulation of feed stream components is therefore almost unavoidable and triggers a rapid flux decline that lowers the process throughput [22,33,34]. Filtration processes are widely used in many industries including wastewater and effluent treatment, food processing, reusable and potable water production and in medical applications such as drug delivery and hemodiafiltration [35,36,37,38,39,40,41,42,43]. Membrane processes can be classified based on nominal pore size in conventional filtration (from 100 to 10 μm), microfiltration (MF) (from 10 to 0.1 μm), ultrafiltration (UF) (from 0.1 μm to ~50 Å), nanofiltration (NF) and reverse osmosis (RO) (both with a pore size down to ~1 Å) and each class is subject to fouling from different species as shown in Figure 1. MF and UF membranes, for example, are particularly suitable for bio-application since their pore sizes are comparable to the average cell or protein dimensions; NF and RO membranes, instead, can be fouled even by small molecules such as salts and ions, since pore diameter corresponds to the dimensions of voids randomly created by the thermal motion of polymer chains [44]. Owing to the wide variety of foulants affecting filtration processes and to the complex interactions involved, membrane fouling has gained a lot of scientific interest, with a large number of studies devoted to the identification of the mechanisms underlying the transient accumulation process [45,46,47,48].

According to Anis et al. [49], membrane fouling forms the third-largest research area in MF with over 600 papers published in the course of 10 years to the date of their work, and such a topic is still receiving attention thanks to the newest trends involving fouling mitigation through membrane modification and innovative investigation techniques aimed at studying the fouling phenomena at a microscopic level [50,51,52,53]. Indeed, the effects governing flux decline generally occur at quite small time and spatial scales, the latter corresponding to the dimension of the pores; studying fouling in micro-sized geometries would therefore allow achieving key insights regarding the interactions between foulants and membrane surface.

### 1.2. Microfluidic Membrane Devices

A technology of great potential for fouling investigation at the micro-scale is microfluidics. In its broadest definition, microfluidics is the science and technology of systems processing low amounts of fluids—between $10^{-18}$ and $10^{-9}$ L—in microchannels with typical dimensions down to micrometers [54,55]. Microfluidics offers a large number of benefits [56]. The most important advantage of microfluidic systems is that the use of small fluid volumes allows reducing the consumption of reagents with a significant decrease in costs, safety risks and waste production, as well as other features including design flexibility, moderate chip cost and the possibility of coupling with microscopy techniques [15,57].

To study membrane fouling, two microfluidic categories can be identified. The first one is also the simplest one, namely the membrane mimicking microfluidic devices (MMM). Such systems reproduce the pore structure of a membrane in an ideal way through parallel straight or constricted channels, round pillars, non-aligned squares or even complex 3D structures to account for higher pore tortuosity [58,59,60,61,62]. Membrane-mimicking devices allow the investigation of the fouling layer evolution in a single pore or in arrays of arbitrary complexity, yet the resolution of many modern fabrication techniques for microfluidic chips establishes a lower limit for pore dimensions and structure design [63]. The second category corresponds to the embedded membrane microfluidic devices; as the name says, these systems consist of chips specifically designed to house a small portion of the membrane [64,65,66,67]. As real membranes can be used, these devices overcome the drawbacks of the previous category, allowing the study of the fouling process at a level of complexity closer to reality; nevertheless, cross-sectional observation of the membrane can be more troublesome compared to MMMs. Embedded membrane microfluidic devices can potentially be useful also to assess the performances of the innovative membrane such as complex-shaped, biocompatible and reinforced ones, as microfluidic modules can be designed ad hoc for specific needs [57,62,68,69].

Many reviews have examined the combination of microfluidics and membranes. A discussion on the application of microfluidic devices to investigate membrane filtration and failure due to particles accumulation can be found in the work of Bouhid de Aguiar et al. [70]; on the other side, Debnath et al. have reviewed several device configurations involved in colloid filtration, whereas other studies focused on microchip fabrication techniques and the implementation of on-chip operations [71,72,73]. These works extensively discuss the advantages and limitations of micro-confined environments to study fouling dynamics, so the aim of this review is not to provide a comprehensive analysis of microfluidics applied to filtration processes, but rather to explore the fouling phenomena through microfluidic systems, assessing the fundamental interactions involved and how microfluidics enables a deeper comprehension of the complicate mechanisms governing the process. The principal mathematical models used to describe the various stages of the phenomena will then be briefly reviewed, in order to determine their advantages and limitations in the comprehension of the still debated aspects of fouling. Lastly, the most important dynamic investigation techniques in which microfluidics plays—or can potentially play—a key role will be discussed, analyzing their working principles and how they have been employed to study fouling.

## 2. Fouling: Stages and Interactions

**Figure 2:**
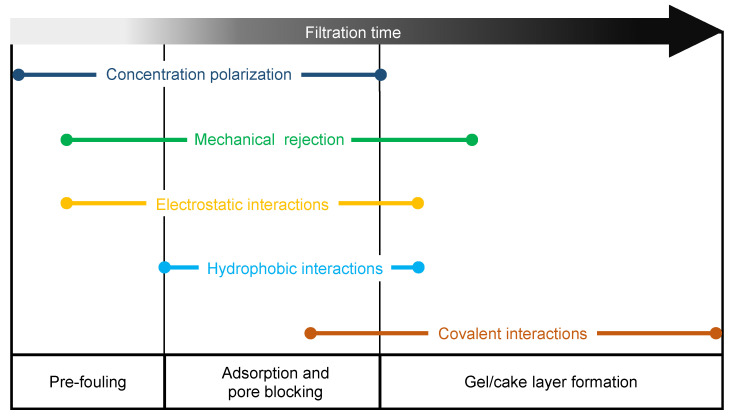
Principal foulant–foulant and foulant–membrane interactions involved in the three stages of fouling.

The fouling process can be generally divided into three stages in series, as depicted in Figure 2: The pre-fouling stage, where foulants migrate from the bulk to the surface proximity; the membrane adsorption and blocking stage, during which pores are gradually clogged, and finally the gel/cake layer stage, in which the membrane is completely covered by foulants [21,74,75]. Each stage is characterized by interactions different in nature between the fouling agents themselves and with the membrane surface. In this section, the three fouling stages will be discussed, assessing the principal interactions involved and their effects in microfluidic systems.

### 2.1. The Pre-Fouling Stage

**Figure 3:**
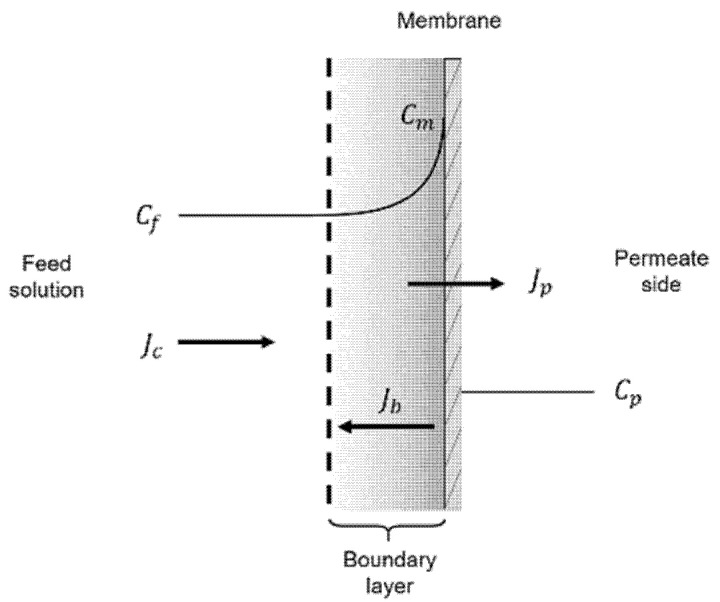
Schematic representation of the concentration polarization phenomenon. $C_{f}$, $C_{m}$ and $C_{p}$, respectively, represent foulant concentration in the feed bulk, at the membrane surface and in the permeate stream, whereas $J_{c}$, $J_{p}$ and $J_{b}$ indicate the convective flux toward the membrane, the permeate flux and the diffusive black flux determined by the concentration difference between the membrane surface and the feed bulk.

The first stage of the fouling process is characterized by foulant migration towards the membrane surface, which starts being physically and chemically conditioned for the sequent stages. Owing to steric hindrance, there is a mechanical rejection of the foulants by the membrane, which causes a rise in concentration near the surface, triggering the so-called concentration polarization phenomenon (CP) (Figure 3). The solutes buildup forms a thin film dominated by diffusive transport, the CP boundary layer, which generates a chemical potential gradient of opposite direction with respect to the permeate flow [44,76]. As a consequence, a species back-flux and an increase in osmotic pressure are determined, with detrimental effects on mass transfer and selectivity. The onset of CP happens as the feed stream comes in contact with the membrane and continues during the whole filtration process, influencing the subsequent fouling stages. However, CP is not irreversible, since interrupting the feed or flowing a clean current would gradually eliminate the back-flux [23,77,78]. In the CP phenomenon, species migrating from the bulk interacts both with the membrane and with other foulants present in the boundary layer. When close to the membrane surface, particles and molecules are subject to long-range electrostatic interactions, which could be either attractive or repulsive according to the carried charges [79]. pH deeply affects the repulsive effects as it impacts on both the sign and number of charges of the functional groups of a molecule. For instance, in the case of proteins, a pH value far from the isoelectric point is responsible for a higher surface charge density, resulting in a strong repulsion from the membrane [80]. The ionic strength of the feed solution plays a role too, due to the possible adsorption of ions [81]. Electrostatic interactions are usually characterized by means of zeta potential measurements [80,82]. When dealing with colloidal particles, foulant–foulant interactions affect the pre-fouling stage as well. Such long-range forces are either of electrodynamic or electrostatic origin and significantly impact the formation and permeability of the CP layer; colloidal particles are indeed subject to repulsive forces arising from the interaction of the electrical double layers surrounding them [83]. Depending on their diameter, particles in the boundary layer can also experience effects such as Brownian or shear-induced diffusion and hydrodynamic lift forces that can help in retarding the onset of CP [84,85]. From simulation studies, Wang et al. showed that nanometric particles are mostly affected by inter-particle interactions, while micrometric ones are predominantly influenced by hydrodynamic lift forces; on the other hand, for particles around 100 nm, none of these interactions plays a major role [86].

**Figure 4:**
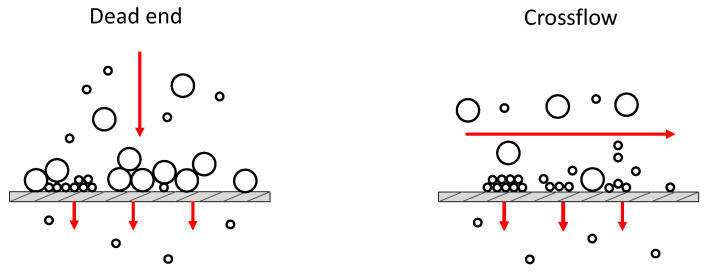
Dead end and crossflow filtration modalities.

**Figure 5:**
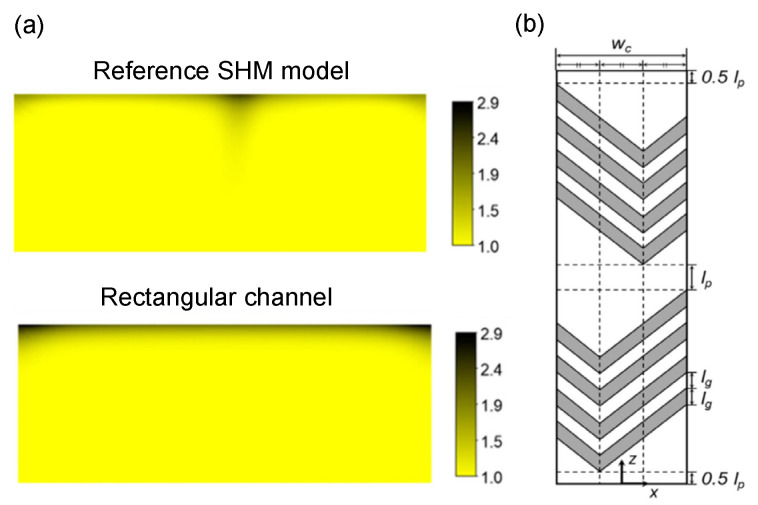
(**a**) Evolution of the concentration distribution in a staggered herringbone mixer (SHM) and in a plain rectangular channel with a permeating wall on top. Color contours represent the dimensionless concentration; (**b**) periodic unit of a staggered herringbone mixer. Adapted from [93].

CP is ubiquitous in all the filtration processes, as it characterizes the first phases of fouling evolution, and its importance depends on the membrane separation process. Nevertheless, most studies have focused on the analysis of the long-term flux decline mechanism, where CP’s importance becomes marginal [46,87]. The role of CP in forward osmosis (FO) processes was investigated by Jiao et al., who characterized the development of the boundary layer using a PDMS embedded membrane microfluidic device [88]. Here, experiments were performed flowing the feed solution tangentially to the membrane surface, in the so-called crossflow operation mode (Figure 4); such configuration is known to be beneficial for fouling prevention as it adds a shear mediated transport back to the feed bulk [89]. Using fluorescence microscopy (Figure 5a), the authors were able to visualize the CP layer and monitor its thickness and permeate flux under various tangential flowrates. From the results, they concluded that CP mitigation becomes insignificant as the feed flowrate increases beyond a certain value.

Kaufman et al. explored the implementation of NF and RO processes in microfluidics, optimizing system design in order to contain CP [90]. Joining experimental results and CFD simulations they studied the role of the feed channel hydraulic diameter, showing that its decrease positively affects the mass transport coefficient, thus mitigating the CP phenomenon. The negative effects of CP on NF processes have also been studied by Completo et al., who showed the lower performances of a microfluidic crossflow NF device in comparison to a centrifugal NF system [91]. The optimization of the chip design for fouling mitigation represents a crucial challenge in microfluidic applications, since the flow in such systems is laminar, without any possible convective mixing [92]. Several studies have focused on the implementation of static elements inside microfluidic channels to induce mixing. Among all the different configurations, ribs and staggered herringbones geometries have been successfully used in the investigation of CP (Figure 5a,b) [77,93].

### 2.2. Membrane Adsorption and Pore Blocking

Adsorption and pore blocking define the second stage of the fouling process: Once foulants have traveled from the bulk solution through the CP layer, they come in contact with the membrane surface and the walls of its pores, where system evolution is governed by surface–foulant interactions [48]. With specific reference to the adsorption phenomena, this stage is usually termed “prompt fouling” and happens on very short time scales compared to the long-term flux decline imputable to the formation, growth and settling of a cake (or gel) layer [22]. In the industrial field, membrane performance are often characterized after the onset of prompt fouling; some membranes are indeed commercially useful only after it takes place [22,94]. Nevertheless, it is principally a negative phenomenon.

Depending on the chemical nature of the species, covalent or non-covalent interactions can occur. Covalent bonds can take place between the functional groups on the foulant particles and the membrane surface; this type of adsorption if affected by the nature of the ligands and by the ion concentration in the feed solution [23,95]. The electrostatic forces, whose mechanisms have already been discussed in the previous section, constitute the first kind of non-covalent interactions that affect the adsorption and pore-blocking stage. In the presence of repulsive interactions, it is possible to individuate a critical flux, as defined by Howell [96], below which no deposition occurs since the drag forces are unable to overcome membrane-foulant repulsion. The first studies on the critical flux in MF and UF have been particularly useful for membrane plant operators, interested in maximizing the permeate flux while reducing cleaning operation frequency [78,97,98]. Recently, Lucas et al. applied microfluidic technologies to characterize the critical flux behavior of ultrathin nanoporous silicon nitride (NPN) membranes in crossflow filtration of concentrated protein solutions [99]. Results showed that the NPN membrane thickness played a key role in terms of fouling mitigation. Higher critical fluxes were indeed achievable thanks to the lower trans-membrane pressure required to achieve the desired fluxes, without leading to the formation of a compacted protein layer on the membrane surface. Van Zweiten et al. studied the effects of trans-membrane flux on clogging dynamics (e.g., the rate of pore blocking) by means of an MMM crossflow filtration device and a solution of polystyrene particles (${\bar{d}}_{p}=2.4\;\mu m$) [100]. According to the authors, the lower clogging time at higher trans-membrane fluxes can be explained by the interplay of two opposing effects: The dependance of particle adsorption probability on its residence time in the pore and the higher viscous drag forces at higher fluxes, which push particles away.

**Figure 6:**
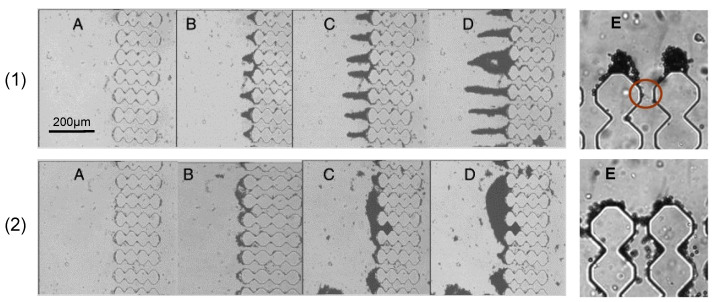
Observation of pore clogging caused by particle deposition over time (from (**A**–**D**)). Two different behaviors can be identified based on PDMS surface properties. Row (1) shows pore blocking in hydrophilic conditions, where particles form dendrites at the pore entrances. Row (2) depicts clogging in hydrophobic conditions; here frequent particle collisions with the wall promote the formation of arches (onset **E**) and the subsequent formation of a cake layer. Adapted from [59].

Hydrophobic interactions constitute the second category of non-covalent interactions and involve van der Waals forces and Lewis acid–base interactions, which also include hydrogen bonding [74]. Membrane-foulant hydrophobic adsorption particularly affects the initial stages of fouling, and its dynamics can be explained considering hydrogen bonding. The presence of a hydrophobic surface in water disturbs the preexisting network of hydrogen bonds of water molecules, increasing the free energy of the system; as a consequence, hydrophobic surfaces will be naturally pushed together so as to reduce the water-contacting interfacial area [101]. Membrane and foulant hydrophobicity is notoriously known to be one of the main contributors to fouling especially in protein solution processing [48,102,103,104,105]. Using a microfluidic approach, Bacchin et al. showed how a small change in surface properties of a poly-dimethylsiloxane (PDMS) has drastic consequences on pore blocking and particle adsorption [59]. In hydrophobic conditions, particles formed arches at pore entrances, leading to the formation of a subsequent cake layer; on the other side, for hydrophilic PDMS, particles tended to settle on the walls between adjacent microchannels, forming dendritic structures without causing a severe pore blockage (Figure 6).

Membrane morphological characteristics, such as pore size and shape, tortuosity, connectivity and surface roughness, are relevant for this stage of fouling as well. Bacchin et al. studied the role of connectivity and tortuosity on pore blocking using a microfluidic separator. Experiments on filtration of mono-sized latex microspheres were performed using three microchannel geometries, namely an array of straight parallel microchannels and two sets of square pillars, the former aligned on three rows, the latter staggered [61]. The feed solution was processed in dead end mode, that is flowing the feed current perpendicularly to the membrane surface [22].

In the most tortuous configuration (e.g., the staggered pillars), particle deposition occurred firstly in the internal spaces of the membrane resulting in a slow evolution of the fouling layers; for connected channels, an intermediate clogging was observed, as the initially captured particles modified the streamlines inside the channels eventually leading to internal blocking. The parallel channels configuration was the least performing, especially at higher velocities, where particles blocked pore entrances by forming arches. Similar dynamics were also observed in membrane experiments, yet not at the pore scale as in Bacchin’s studies [106]. Microfluidics has also been adopted to investigate blocking behavior as a function of pore shape and size [107]. Massenburg et al. showed the positive effects of converging microchannels on the reduction of clogging times: The presence of a constriction induces higher fluid velocity and therefore higher shear rates, which prevent particles from attaching to channel walls [108]. As discussed, membrane morphology impacts adsorption and pore blocking dynamics; however, such phenomena, in turn, affect membrane structure, resulting in a detrimental feedback mechanism that eventually leads to the formation of a cake/gel layer. In fact, hydrophobic adsorbed foulants narrow pores, enhancing the mechanical capture of species and causing an acceleration in concentration build-up at the membrane surface.

### 2.3. The Gel/Cake Layer Formation

The final stage of the fouling process is characterized by the formation of a filter cake on the membrane surface, acting as a shield and thus providing an additional resistance to permeate flux. According to recent theories, the formation of a gel layer happens as soon as the foulants concentration at the membrane surface reaches a critical value, namely the gel concentration, which marks a phase transition point [109,110]. Indeed, below the gel concentration, the system behaves like a true solution, while above, it is characterized by an ordered phase having a cake type behavior [111]. Depending on water and foulant concentration, one can distinguish between the gel and cake layer; however, the two definitions often refer to the same species [46,112,113]. The gel/cake layer stage is also referred to as cumulative fouling because of its time-dependent nature: After the initial formation due to the high concentration at the membrane, the gel layer keeps growing, fed by the subsequently arriving species from the bulk. Moreover, under the effect of the feed stream, the cake can undergo compaction and structure reorganization, eventually decreasing permeate flux until a stationary value is reached [22].

**Figure 7:**
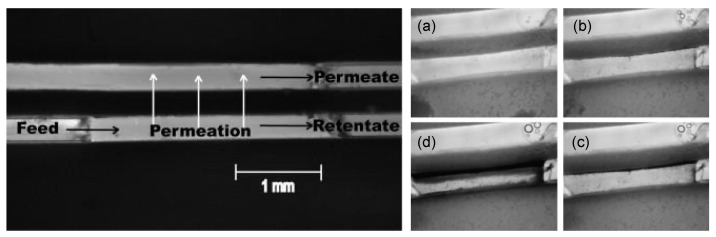
On the left, a microfluidic filtration device allowing side observation of the membrane developed by Ngene et al. Pictures on the right show the temporal evolution (from **a**–**d**) of the cake layer, which can be clearly distinguished in (**d**). Adapted from [67].

This fouling stage is dominated by foulant–foulant cohesion, as species approaching the membrane will interact with the already deposited ones, contributing to the growth of the filter cake. In particular, covalent interactions are the main forces leading the gel layer formation. In filtration processes, common gelling foulants are organic substances, e.g., proteins, polysaccharides and humic acids, typically carrying carboxyl, hydroxyl and phosphoric groups, which can be subject to metal-organic complexation with multivalent ions such as calcium and magnesium [46,74]. In this way, molecules in the gel matrix are crosslinked in a three-dimensional network and the fouling layer reaches macroscopic electro-neutrality [114]. Despite its lower thickness and higher porosity compared to a cake layer formed by particles or sludge flocks, the gel layer presents a contradictory high specific resistance; however, explanations of this behavior are still debated [46,114]. As regards non-covalent interactions, their effects principally influence the layer’s structure in terms of porosity, compactness and permeability. In a recent study, Mokrane et al. investigated the microstructure of the cake layer formed upon the filtration of a colloidal suspension in an MMM device consisting of an array of parallel straight microchannels [115]. Apparently, changes in ionic strength and pressure did not affect the global porosity of the cake layer; nonetheless, a local study revealed heterogeneity in the clog’s structure. Cake porosity was indeed higher nearby the pore entrances, while it was lower far away; furthermore, higher ion concentration in the suspension resulted in smaller colloidal crystals and in more organized structures. These findings allowed the authors to develop a phase diagram concerning foulant–foulant repulsive interactions and hydrodynamic forces. The effects of the latter on the cake growth were also studied by Ngene et al., who developed a filtration microfluidic device, shown in Figure 7, that allowed for side observation of the cake layer formation on the membrane [67].

Foulant–foulant interactions play an important role also in systems containing both organic and inorganic foulants, such as proteins and silica nanoparticles, which can have a synergic effect on cake formation [116]. The dynamics of the cake/gel layer stage are also influenced by particle deformability and compressibility. In fact, even if larger than the pore, soft particles are able to pass through the membrane by deforming and deswelling. Such foulants are common in several industrial applications, e.g., wastewater treatment and emulsion filtration [117,118]. The behavior of a soft microgel in a microfluidic filtration system and in a centrifugation one was investigated by de Aguiar et al. [119]. While at low pressure, pores were blocked immediately, microgel particles were more prone to deformation at higher pressures, clogging pores deeper in the structure of the model membrane.

The cake deformation recovery process was assessed via centrifugation experiments, which allowed an irreversible compression impossible to reach in filtration tests. Pore geometry was shown to play a role in cake formation, too. In cases of soft particle filtration, it is important to note that pore size and membrane cut-off are not reliable parameters when it comes to process efficiency evaluation; indeed, further factors such as particle mechanical properties have to be taken into account.

### 2.4. Biofouling

**Figure 8:**
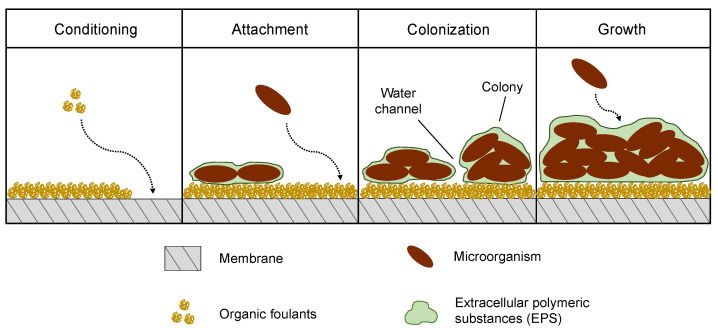
Schematic representation of biofouling evolution stages.

The unwanted deposition and growth of microorganisms on surfaces characterize the so-called biofouling phenomenon [120]. This process generally follows a series of stages, schematically represented in Figure 8, which shares some similarities with the already discussed ones. Indeed, biofouling usually starts with a conditioning phase where organic foulants accumulate and adsorb on the membrane surface; once microorganisms have attached to the membrane, however, colonies can form and proliferate, eventually forming a mature layer that can be detached by the shear forces the feed flow exerts [121].

Microorganism colonization of a surface is actually a complex process, in which various phenomena take place at different time and length scales; the interactions of bacteria, fungi or algae with the membrane causes the formation of a complex matrix, which hosts microorganisms and is made of nutrients and biological waste products, the extracellular polymeric substances (EPS), that are very prone to gelling [122]. This constitutes a major issue during cleaning procedures. The common response to biofouling is disinfections, which consists of killing the microorganisms that, however, may still remain attached to the membrane, becoming a nutrient supply for the survived ones [123]. In this way, the exponential proliferation would restore the colony in very short times, resulting in useless disinfection. It is therefore necessary to ensure the complete removal of the biofilm by overcoming the adhesive and cohesive forces—i.e., hydrophobic interactions, hydrogen bonding, entanglements—which bind it to the membrane surface and are provided by di EPS. The most common removal procedures involve hydraulic and pneumatic cleaning [123].

Biofilm formation is affected by several biological factors, including cell physiology, mechanical properties and physicochemical factors, such as hydrodynamic conditions and membrane morphology [50,122]. These elements impact the film structure, leading to uncommon fouling phenomena such as the formation of filamentous structures, termed streamers, downstream of the membrane pores [124]. Microfluidics has been proven to be a useful tool for the investigation of bacterial streamers in membrane filtration. Marty et al. investigated the formation of streamers and the effect of pore size and structure, filtration mode and flowrate on the filtration of an *Escherichia coli* suspension in an MMM device [125]. The streamer formation process is characterized by three steps. Initially, tiny filaments start to adhere to the channel entrances without interfering with bacterial accumulation; after approximately 1 h, multiple filaments interact to form a network that captures more cells, eventually forming larger bacterial streamers. This process is deeply affected by pore tortuosity, rather than connectivity. In fact, of the three geometries used by the authors, the staggered pillars one exhibited the most severe filament formation, due to the numerous changes in flow directions. These microfluidic configurations were the same as in the work of Bacchin et al. [61], discussed in Section 2.2. Membrane adsorption and pore blocking. In addition, streamer build-up was enhanced by smaller pores and lower flowrates; on the other side, crossflow filtration mode appeared to promote streamer formation, although at different magnitudes depending on the pore structure.

## 3. Fouling Mathematical Modeling

The evolution of fouling during a filtration process is typically monitored by means of techniques that rely on the measurement of parameters such as averaged permeate flux, solute rejection and pressure drop across the filtration cell. In order to assess the various stages of the phenomena, various mathematical models have been proposed to analyze data in both dead-end and crossflow filtration modes. Specific equations have been developed for constant pressure and constant flux, the former being characterized by a flux decrease over time due to fouling, whilst in the latter, an increase in pressure can be observed. In this section, different mathematical models for membrane fouling interpretation will be reviewed briefly, with particular attention to constant pressure filtration.

### 3.1. Resistance-in-Series Model

The resistance-in-series model derives from Darcy’s law and is based on the concept of total resistance, which includes contributions from the intrinsic membrane resistance and from the resistance generated by fouling development [126]. Assuming constant pressure and applying Darcy’s law [127]:

(1) $$J=\frac{\Delta p}{\eta \;R_{tot}}$$

where J is the permeate flux ($m^{3}m^{-2}s^{-1}$), Δp is the transmembrane pressure (Pa), η is the viscosity of the permeate (Pa s) and $R_{tot}$ is the total resistance ($m^{-1}$). $R_{tot}$ can also be expressed as the sum of three contributions, namely $R_{m}$, $R_{r}$ and $R_{ir}$:

(2) $$J=\frac{\Delta p}{\eta \;\left(R_{m}+R_{r}+R_{ir}\right)}$$

where $R_{m}$ is the hydraulic resistance of the clean membrane, while the other two terms respectively represent the resistance caused by reversible phenomena such as CP ($R_{r})$ and the one caused by irreversible phenomena, e.g., adsorption and permanent external or internal pore blockage ($R_{ir}$). $R_{m}$ can be calculated by performing pure water flux experiments (i.e., in absence of foulants); to calculate $R_{ir}$, a second pure water flux experiment must be performed after carefully rinsing the membrane subsequently to the filtration of the desired feed. A second pure water flux ($J_{w}^{\prime }$) can thus be measured and $R_{ir}$ calculated as follows:

(3) $$R_{ir}=\frac{\Delta p}{\eta_{w}\;J_{w}^{\prime }}-R_{m}$$

indicating with $\eta_{w}$ the viscosity of pure water. $R_{r}$ can be finally evaluated by subtracting the irreversible and membrane resistances to the total resistance. Equations (1)–(3) can be used without further modification for both dead end and crossflow filtration mode.

By evaluating the separate terms contributing to $R_{tot}$, the resistance-in-series model allows one to study which terms play a key role in the flux decline, giving useful information about whether a filtration process is controlled by reversible or irreversible fouling and thus which cleaning mechanism could be the most appropriate for fouling mitigation [128]. Moreover, from the concavity of $R_{tot}$ as a function of time, it is possible to distinguish between external and internal fouling, or rather if the adhesion of foulants takes place on the membrane surface (downward concavity) or within the pores (upward concavity) [129,130]. Nevertheless, the resistance-in-series model does not provide specific information about the fouling mechanisms. Indeed, from the evaluation of $R_{tot}\left(t\right)$ it is only possible to tell where the foulants are settling, yet how their deposition happens (e.g., complete blocking of the pore entrance or cake formation) is not fully known.

### 3.2. Blocking Filtration Laws and Cake Filtration Model

**Figure 9:**
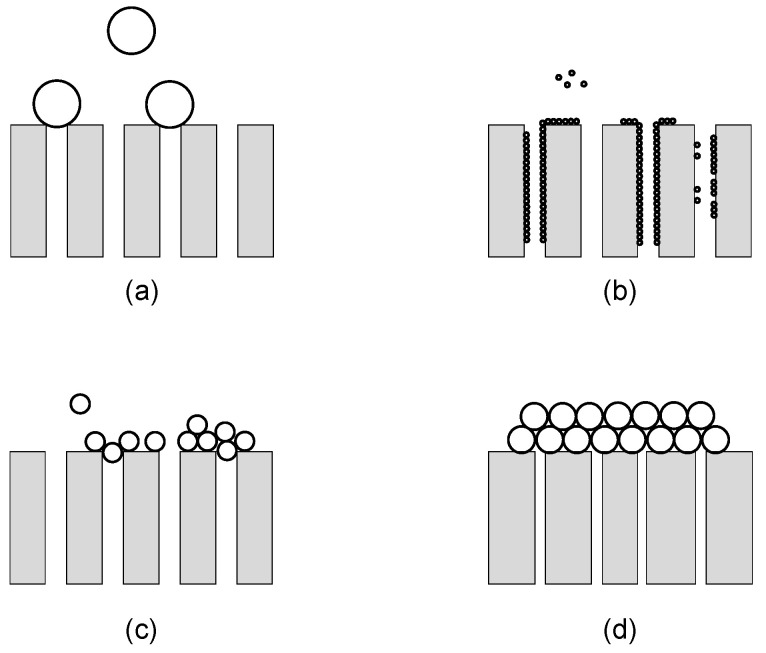
Hermia’s pore blocking models illustration: (**a**) Complete blocking; (**b**) standard blocking; (**c**) intermediate blocking; (**d**) cake filtration.

The blocking filtration laws and the cake filtration model represent a very useful tool for the interpretation of the physical mechanism governing the blockage of pores over time. The model takes into account the dependence of the filtration behavior on the ratio of the particle size to the pore opening one and consists of four different fouling mechanisms: Complete blocking, standard blocking, intermediate blocking and cake filtration, as represented in Figure 9 [131].

The filtration laws were first proposed by Hermans and Bredée for constant pressure filtration and subsequently reanalyzed by Grace and Hermia, who condensed the laws for the four mechanisms into a single differential equation by adjusting two parameters characteristic of the specific fouling behavior [132,133,134]:

(4) $$\frac{d^{2}t}{dv^{2}}=K_{DE}{\left(\frac{dt}{dv}\right)}^{n}$$

where t is the time (s) and v is the filtrate volume ($m^{3}$). $K_{DE}$ and n are constants related to the fouling behavior, the former’s physical meaning and the latter’s value depends on the mechanism. Equation (4) can also be expressed in terms of flux J=dv/dt [131]:

(5) $$\frac{dJ}{dt}=-K_{DE}J{\left(J\right)}^{2-n}$$

A further development of Equations (4) and (5)—which can only be used for constant pressure dead end filtration—was made by Field et al., who adapted Hermia’s blocking filtration laws to constant pressure crossflow filtration by adding a term that represents convective removal:

(6) $$-\frac{dJ}{dt}=K_{CF}\;\left(J-J^{*}\right)\;J^{2-n}$$

where $J^{*}$ can be considered as a critical flux that should not be exceeded in order to avoid fouling and is usually assumed as the steady-state flux value (J(t→∞)), while $K_{CF}$ and n are the parameters correlated to each model [126,128]. Table 1 summarizes the model equations for constant-pressure dead-end and crossflow filtration.

The complete blocking model is characterized by n=2 and is based on the assumption that, considering a membrane with parallel pores of constant diameter and length, each foulant particle arriving at its surface completely seals the entrance of an open pore without depositing over other particles. The permeate flux through unblocked pores is thus unaffected and its reduction over time is equal to the decrease in membrane area available for filtration; as a matter of fact, the parameter $K_{CPB}$ represents the membrane surface blocked per unit of total volume permeated and unit of initial membrane surface porosity [129,135]. This fouling behavior is typical of systems involving particles whose size is bigger than the pore diameter.

Differently from the complete blocking model, where particle deposition occurs on the membrane surface, the standard blocking model considers the deposition and adsorption of foulants inside the membrane pores due to the irregularity of pore passages. As a consequence, pore diameter decreases proportionally to the permeate volume. Foulants smaller than the pore size are the main responsible of this fouling mechanism, which is described by n=3/2 and its constant $K_{SPB}$ represents to the volume of solid retained per unit of filtrate volume, membrane thickness and inverse surface porosity [135]. It is worth noting that, as fouling takes place on the inside of the pores, in crossflow filtration, flux reduction does not depend on the crossflow velocity and the steady-state permeate flux J(t→∞) is zero [136]. The equation describing this model for dead-end and crossflow filtration is therefore the same [129].

According to the intermediate blocking model (n=1), pores are not necessarily blocked by one particle, and the probability that the settlement takes place on an already deposited particle must be taken into consideration; consequently, during filtration, the clean membrane surface diminishes along with the probability of a particle blocking a pore [98]. This model well describes systems where the foulant size is similar to the pore size and, therefore, pore entrances are obstructed but not completely blocked. The physical meaning of the parameter $K_{IPB}$ is similar to the one characterizing the complete blocking model.

Because of its assumptions, the cake filtration model is usually considered apart from the previously described models, which are categorized as pore blocking models. Indeed, in cake filtration, foulants deposit on the membrane surface without entering the pores and a filter cake grows throughout the filtration process adding additional resistance to the permeate flow [131]. In the cake filtration model, n=0 and the constant $K_{CLF}$ is related to both the characteristics of the cake and those of the clean membrane [98].

### 3.3. Combined Models

To understand the fouling mechanisms that interest a filtration process, permeate flux data as a function of time are usually inferred using only one of the discussed blocking filtration laws or cake filtration model for the entire range of data [45,60]. Although such a procedure can lead to good interpretation, it typically represents the main disadvantage of the presented models. Indeed, fouling is a three-stage process (i.e., pre-fouling, pore blocking and cake formation stages) in which complex mechanisms take place, caused by the presence of particles of different sizes, which might interact with both the membrane and other particles. Moreover, real membranes usually present a pore size distribution and a complex morphology far from that of the parallel straight cylinders structure assumed by the models. A succession of pore blocking mechanisms hence occurs, where transitions between consecutive stages are gradual and happen over small time intervals [129]. Consequently, data fitting can be affected to the point that none of the mechanisms is able to properly explain the flux reduction due to fouling [129]; for this reason, combined models were developed when multiple mechanisms dominate the evolution of fouling.

An approach to data interpretation considering multiple fouling mechanisms is that of inferring the whole set of flux data over time with each one of Hermia’s models (single-stage Hermia model) and analyzing which one gives the best fit for a specified time interval. Such a procedure was adopted by Brião et al. to evaluate the fouling behavior of ultrafiltration membranes, where the resistance-in-series model was also used to understand where the fouling happened preferentially [126]. A similar methodology was implemented by Choobar et al., who used a multistage Hermia model capable of giving information about the dominant mechanism at different times by only fitting a certain interval with the most appropriate mechanism [45]. The general equation of such a model is Equation (7):

(7) $$J_{Multistage\;model}=a\;J_{CPB}+bJ_{IPB}+cJ_{CLF}$$

where a, b, c are factors varying with operating conditions (e.g., TMP and crossflow velocity) while $J_{CPB}$, $J_{IPB}$ and $J_{CLF}$ are, respectively, the permeate flux connected to the complete blocking, the intermediate blocking and the cake filtration. The proposed model was obtained by first inferring the flux data with a single-stage Hermia’s model, which revealed that standard blocking had the lowest overall fitting accuracy and was therefore discarded in the multistage model. The single-stage model-fitting procedure also allowed to identify the dominant mechanism at different times, useful for the subsequent application of the multistage model.

Ho and Zydney developed a combined model for protein fouling, which accounts for both initial pore blocking and subsequent cake layer growth by providing a smooth transition between the two mechanisms without the need for multiple mathematical expressions [137]. The model was derived for dead-end constant-pressure filtration and considers the volumetric flowrate permeating the membrane to be equal to the sum of two flowrates, the former related to open pores while the latter to the blocked ones. The filtrate flowrate at any given time is hence calculated as follows:

(8) $$Q\left(t\right)=Q_{0}\left[\exp\left(-\frac{\alpha \Delta pC_{b}}{\eta R_{m}}t\right)+\int_{0}^{t}\frac{\alpha \Delta pC_{b}}{\eta (R_{m}+R_{p})}\;\exp\left(-\frac{\alpha \Delta pC_{b}}{\eta R_{m}}t_{p}\right)dt_{p}\;\right]$$

where Q and $Q_{0}$ respectively are the volumetric flowrates ($m^{3}s^{-1}$) at a given time t and the initial volumetric flowrate through the clean membrane, Δp is the transmembrane pressure (Pa), α is a pore blockage parameter ($m^{2}\;{\mathrm{kg}}^{-1}$) related to the mass of an aggregate blocking a given area of the membrane, $C_{b}$ is the bulk concentration ($g\;l^{-1}$), $t_{p}$ is the time at which a particular region is first blocked by a protein aggregate (s), $R_{m}$ is the resistance of the clean membrane ($m^{-1}$) and $R_{p}$ is the resistance of the protein deposit ($m^{-1}$) given by Equation (9):

(9) $$R_{p}=\left(R_{m}+R_{p0}\right)\sqrt{1+\frac{2f^{\prime }R^{\prime }\Delta pC_{b}}{\eta {\left(R_{m}+R_{p0}\right)}^{2}}\left(t-t_{p}\right)}-R_{m}$$

where $R_{p0}$ is the resistance associated with a single protein aggregate ($m^{-1}$), $R^{\prime }$ is the specific protein layer resistance ($m^{-1}$) and $f^{\prime }$ is the fraction of proteins that contribute to the growth of the deposit. The smooth transition between the initial fouling stage and the cake formation provided by the model derives from the assumption that settled aggregates allow a small amount of fluid to flow through pores, with the hydraulic resistance of the fouled areas of the membrane increasing in time because of the proteins transported to the surface. The model thus considers the spatial inhomogeneity of the protein layer, with the firstly fouled regions of the membrane opposing higher resistance to the permeate flow.

**Figure 10:**
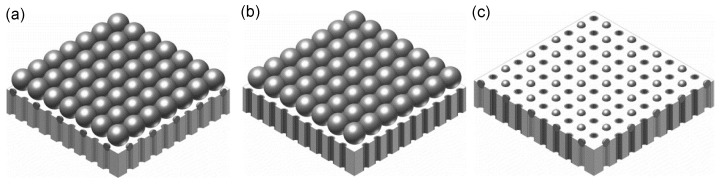
Bolton’s combined models: (**a**) Cake filtration—complete blocking; (**b**) cake filtration—standard blocking; (**c**) complete blocking—standard blocking. Adapted from [138].

A set of five models accounting for the combined effect of different fouling mechanisms was proposed by Bolton et al., instead [138]. The authors derived the model equations from Darcy’s law for both constant pressure and constant flow dead end filtration. The most effective model among the proposed ones is the complete blocking–cake filtration one (Figure 10a), which assumes that cake formation and complete pore blockage happen simultaneously and independently. Here the cake forms on unblocked membrane areas whereas complete blocking can occur where the cake has previously formed.

Therefore, flux reduction is caused by a decrease in the area available for filtration due to complete blocking or by an increase in resistance related to cake growth. The assumptions of this model make it suitable in the presence of distinct foulant species, for example large particles settling on the membrane surface and smaller ones permeating through the cake and blocking membrane pores. In practice, however, the two mechanisms are usually connected as blocked pores could lead to the buildup of a cake; on the other side, an already formed cake layer could prevent a subsequent pore blockage. In the combined cake filtration–standard blocking model (Figure 10b), the available filtration area does not decline over time, as pores are fouled on the inside rather than blocked at the entrance. This model can be useful in the presence of particles smaller than the pore size and much larger ones, as the former will penetrate inside the pores whereas the latter will settle on the membrane surface constituting an additional hydraulic resistance (i.e., cake resistance). As for the complete blocking–cake filtration model, although the two mechanisms are supposed to act independently, they can actually influence one another. Indeed, the presence of a cake could prevent small particles from permeating inside the pores, while constricted pores could enhance particle deposition on the membrane surface. The other three models involve the combination of intermediate blocking with cake filtration or standard blocking and that of complete with standard blocking. As pointed out by the authors, the five models are less physically detailed than the combined model proposed by Ho and Zydney and their use for the estimation of physical parameters is limited; however, the reduced numerical complexity make them easier to implement [137,138]. A complete overview of the models’ equations in terms of permeate volume as a function of time for constant pressure filtration can be found in Table 2.

More recently, Bolton’s complete blocking–cake filtration model was improved by Hou et al. [139]. The authors proposed a new combined model, which considers an initial time interval where complete blocking and cake filtration acted together, followed by a long-term flux decline related to the dominance of the latter. A steady frontal membrane area K ($m^{2}$) was introduced, representing the available membrane frontal area left when the fouling mechanism switches from combined complete blocking–cake filtration to individual cake filtration. Moreover, the transition point between the two mechanisms was determined under various process conditions. According to this model, flux decrease as a function of time is expressed by Equation (10):

(10) $$J=\frac{J_{0}\left(\left(1-K\right)\exp\left(\frac{-K_{b}}{K_{c}J_{0}{}^{2}}\left({\left(1+2K_{c}J_{0}{}^{2}t\right)}^{1/2}-1\right)\right)+K\right)}{{\left(1+2K_{c}J_{0}{}^{2}t\right)}^{1/2}}$$

where $J_{0}$ is the initial permeate flux ($m^{3}m^{-2}s^{-1}$) while $K_{b}$ and $K_{c}$ are the complete blocking ($h^{-1}$) and cake filtration constant (${h\;m}^{-2}$) of Bolton’s combined model.

## 4. Dynamic Investigation Techniques

As previously discussed, fouling evolution is usually monitored through flux and pressure measurements. Despite the fact that mathematical modeling represents a useful approach to characterize the various fouling mechanisms, such parameters only enable an indirect evaluation of all the complex phenomena happening at the microscale, and few pieces of evidence are provided on foulant concentration and distribution over the membrane. In addition, CP and cake compression can affect both permeate flux and transmembrane pressure (TMP), resulting in possible data miscomprehension. The time resolution of conventional methods represents a drawback too: CP layer development, adsorption and pore blocking are indeed very fast processes whose characteristics may not be fully elucidated by simple gravimetric experiments and pressure readings.

It is therefore challenging to assess each stage of the fouling phenomena with such approaches, and new process-oriented methodologies are currently being explored [140]. Specifically, in situ real-time monitoring techniques are very powerful tools to investigate the dynamic development of fouling. In a recent paper, Rudolph et al. reviewed several of such techniques available for the monitoring of membrane fouling in the biotechnology, biorefinery and food sectors, enlightening their strengths and weaknesses [141]. Applications of in situ techniques to CP monitoring were instead reviewed by Chen, Li and Elimelech [109]. A deeper comprehension of the microscopic events involved in the evolution of fouling has been enabled by the coupling of in situ dynamic monitoring techniques with microfluidics. As a matter of fact, the micro-confined environments provided by the latter allow one to observe phenomena such as pore occlusion and cake growth with high spatial resolution. In the next section, different investigation techniques in which microfluidics represents a potentially valuable tool for the study of the fouling phenomena will be reviewed.

### 4.1. Direct Microscopic Observation

**Figure 11:**
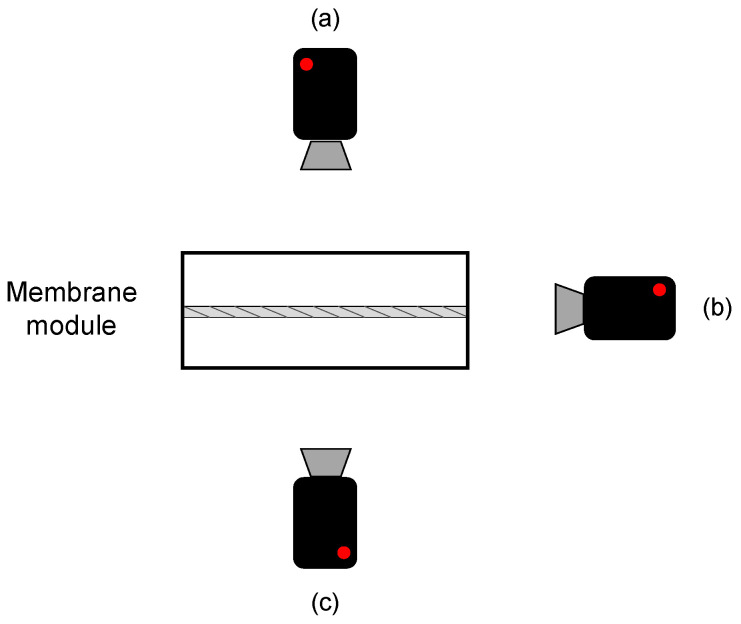
Schematic of direct visual observation: (**a**) DOSM; (**b**) DVO from side view; (**c**) DOTM.

Direct in situ observation (DO) of foulant deposition on the membrane surface and inside the pores constitutes a simple, non-invasive and low-cost technique for membrane fouling monitoring. DO can be generally classified into two categories: Direct visual observation (DVO) and direct observation through the membrane (DOTM) (Figure 11) [140,141].

In the former, the membrane is observed from above its surface on the feed side, allowing the visualization of fouling as far as the feed current is transparent enough for light to pass; DVO is also known as direct observation of the surface of the membrane (DOSM) and can be performed even observing the membrane from a side view, enabling cake layer thickness measurements and observation of the inside of the pores [142]. On the other side, DOTM are only possible if membranes are transparent under process conditions and are limited to the monitoring of the first stages of the process, after which the formation of an opaque layer of foulants impedes any observation [67].

The use of microscopy as support for DO enables the visualization of the events involved in the fouling phenomena that take place at the microscopic scale. In such a way, it is possible to determine foulant deposition dynamics as well as deposit morphology and how these are affected by process parameters (i.e., TMP, feed flowrate, membrane properties); this information can be thus related to flux and pressure data, allowing a detailed comprehension of the fouling mechanisms. Among the various microscopy techniques, light-microscopy finds extensive usage for in situ real-time DO owing to its versatility [140]. In particular, among the many types of light-based techniques, the coupling of microfluidics with bright-field microscopy, fluorescence microscopy and confocal laser scanning microscopy (CLSM) represents the most used and effective methodologies to investigate the fouling phenomena.

#### 4.1.1. Bright-Field Microscopy

Bright-field microscopy is the most common imaging technique in light microscopy [143]. Modern bright-field microscopes are compound microscopes using multiple lenses systems to form a dark image on a bright background. Indeed, in such apparatus the illuminating beam is a solid cone of light provided by an illuminator located below or above the stage (respectively in upright or inverted microscopes) and focused on the specimen by a condenser lens to maximize illumination. The final image is produced by the consecutive magnifications of the objective lenses (placed near the specimen) and the ocular and can be viewed directly or captured via a digital camera (e.g., digital video microscopy) [144]. Especially for fouling investigations, high-speed cameras constitute an essential tool to monitor the phenomena happening at very short times, such as foulants deposition and pore clogging [145,146].

**Figure 12:**
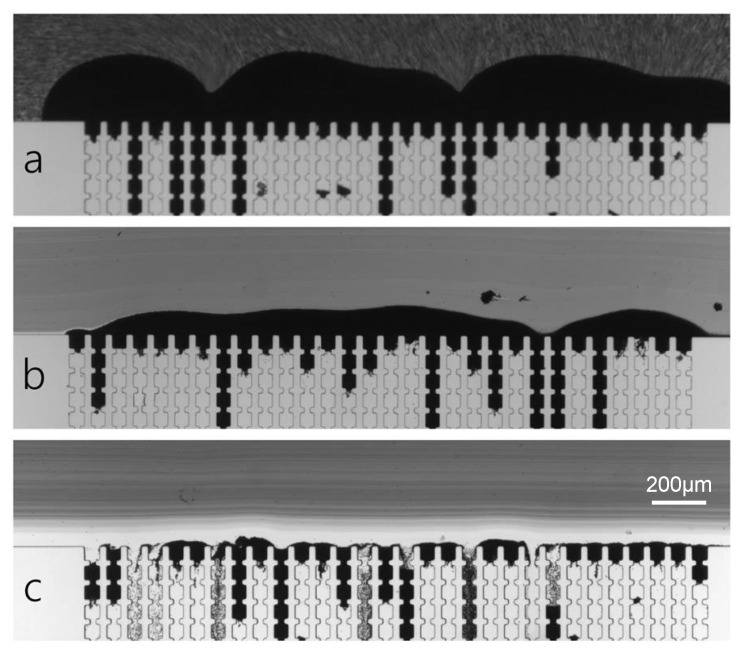
Cake layer formation during (**a**) dead-end and crossflow filtration at 0.219 mL/min and 0.395 mL/min (**b**,**c**). Figures clearly show how particle deposition is influenced by the filtration mode. Black regions indicate the presence of a higher particle concentration. Adapted from [100].

The coupling of bright-field digital video microscopy and microfluidic systems was successfully used by Wyss et al. to study the clogging mechanisms at a single-pore level [147]. In their work, a microfluidic PDMS device consisting of a single wide channel followed by an array of parallel, narrow channels, was employed to analyze the clogging events due to the flow of polystyrene particle suspensions (Figure 12). An additional degree of tortuosity was added by a set of constrictions along the length of each channel. Particle concentration was monitored through image intensity; in fact, the presence of darker regions corresponded to higher particle concentrations and the packing degree of the clogs. Results indicated that neither the feed flowrate nor the particle volume fraction influences the clog formation, which exclusively depends on a critical number of particles flowing through the pore. Moreover, the authors presented a model that accounts for the scaling of such critical particle numbers with the ratio of the pore to particle size. Similar findings were obtained by Dersoir et al., who studied the effects of pore geometry, confinement, hydrodynamic conditions and ionic strength of the solution on the formation of a clog in a single pore [148]. 

However, it appeared impossible to determine the exact clog position and dimensions through bright-field images, as the particle density was too high. Bright-field microscopy has several drawbacks indeed, among which its maximum resolution of about 0.2 μm, which limits its application to micron-sized or smaller foulants [140,143].

Many of the works involving the pairing of bright-field microscopy and microfluidics to study the fouling phenomena make use of MMM devices, which, for construction reasons, allow real-time direct observation of the process from a side view [59,61,100,108,119,147,148]. An application of DO from above can be found in the work of Warkiani et al., who developed a microfluidic embedded membrane device to study the fouling mechanism of micron-sized particles in isopore filters at a macroscopic level [149].

#### 4.1.2. Fluorescence Microscopy

According to IUPAC, fluorescence is the emission of light that intersects particular substances after the absorption of light or other electromagnetic radiation [150]. The key property that makes fluorescence such a powerful tool for visual investigations resides in the so-called Stokes shift, that is, the difference between the emitting and exciting radiation wavelengths, the former being generally higher than the latter. Indeed, blocking the exciting light by means of an optic filter, it is possible to observe fluorescent objects on a dark background with very high contrast. Molecules that undergo fluorescence are called fluorophores and are characterized by a small energy difference between their ground and excited state orbitals, so that even low-energy photons can be used to excite the electrons in the outermost orbitals. Although many natural substances (e.g., chlorophyll) are intrinsically fluorescent, synthetic compounds are usually preferred for labeling because of their better performances [143].

Most of the modern fluorescence microscopes rely on an epi-illumination mode, in which microscope objectives not only image and magnify the specimen, but also act as condensers that focus the light illuminating it. As the paths of the exciting and the emitted lights overlap, such microscopes make use of dichroic mirrors to separate the two. Such filters are engineered to transmit longer wavelengths, belonging to the emission spectra, while reflecting the shorter ones that characterize the excitation light. Dichroics are commonly used with two additional filters, the excitation and the barrier ones, which respectively narrow the exciting wavelengths and the ones belonging to the light going from the sample to the detector. As for bright-field ones, fluorescence microscopes can be supported by a digital camera, which besides the previously described advantages, allows better visualization of dimmed images thanks to the possibility of adjusting the exposure time of the sensor [143].

**Figure 13:**
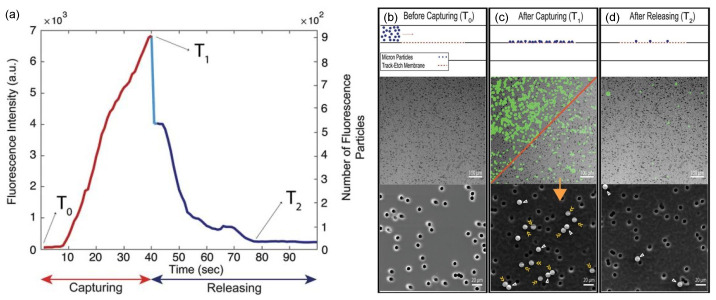
Results from the microscale experiment carried out by Dehghani et al. on microporous track-etched membranes: (**a**) Fluorescence intensity and the number of particles against time plot, showing an increase in the intensity signal and the number of particles during the capturing step and a decrease during the releasing one; (**b**–**d**) TFAC process stages including fluorescence microscopy images (middle) and SEM images (bottom). Adapted from [152].

Real-time observations through fluorescence microscopy were performed by Neeves et al., who monitored platelet aggregation in a membrane-based microfluidic device designed to control the flux of platelet agonists into flowing blood [151]. Time-lapse fluorescence video microscopy was instead used by Dehghani et al. to study pore blocking and adsorption dynamics in a microfluidic device, aimed at the isolation of species such as extracellular vesicles from biological fluids through the so-called tangential flow for analyte capture method (TFAC) [152]. Employing fluorescent particles as model target species to be separated, the authors assessed the efficiency of each stage the TFAC method, namely capturing by crossflow filtration, cleaning with a buffer and releasing via backflushing, for both micro and nanoporous track-etched membranes. Particle deposition in each stage was indeed examined by measuring fluorescence intensity over time (Figure 13).

Fluorescence microscopy was demonstrated to be a helpful tool in the investigation of fouling and sorption mechanisms of proteins. Due to their small dimensions, biological macromolecules such as proteins are indeed impossible to observe through bright-field microscopy, whereas with the help of fluorescent labeling, their clusters and even single molecules can be observed or at least their position and concentration on the membrane surface determined [140]. In recent work, Bacchin et al. combined fluorescence and permeability measurements to study the fouling mechanisms and the adsorption kinetics of bovine serum albumin (BSA) and α-lactalbumin (LALBA) [153].

The real-time in situ observations and flux measurements were allowed by a membrane embedded microfluidic device. Monitoring the changes in fluorescence signal from the retentate side of the membrane surface during filtration showed good agreement between signal and permeate flux values over time. Indeed, the same regimes were observed: The initial flux drop corresponded to a sharp increase in fluorescent signal, while during a second period, a slower flux decline matched with moderate signal variations, eventually reaching steady values. Signal intensity is also correlated to protein concentration, therefore allowing its direct evaluation, useful for sorption kinetic modeling.

Fluorescence tracking during LALBA filtration pointed out one of the main limitations of such microscopy technique, quenching, which is a reversible loss of fluorescence due to noncovalent interactions between a fluorophore and its molecular milieu [154]. As a matter of fact, differently from BSA, LALBA can adopt alternative conformation and remain in a stable, partially denatured state, which can be induced both by flow conditions (e.g., flowrate and TMP) and membrane pore size [155,156]. The principal drawback of fluorescence microscopy is the bleaching phenomenon. Although, theoretically, fluorophores can be cyclically excited infinite times, their usage is generally limited due to the progressive permanent fade of fluorescent signal. There are several strategies to reduce bleaching. First of all, it is important to keep samples in the dark when not in use and to utilize just the right amount of light needed for observations; moreover, high-quality optical filters can decrease bleaching by providing an efficient passage of the emitted wavelengths. As previously mentioned in Section 2.1. The pre-fouling stage, fluorescence microscopy also represents a useful method to investigate concentration polarization [88]. The presence of fluorescent components, indeed, allows the visualization of the concentration gradient across the retentate side, otherwise impossible with bright-field microscopy.

#### 4.1.3. Confocal Laser Scanning Microscopy

Confocal laser scanning microscopy (CLSM) is an optical imaging technique, which allows a high-quality visualization of a three-dimensional specimen at different depths, with better axial resolution and contrast compared to conventional light microscopy techniques. Its performances are accomplished by actively suppressing out-of-focus light thanks to the use of point illumination, provided by lasers, and pinholes [157].

Although, in theory, confocal microscopes can be used for any type of microscopy, their most common application is for fluorescence microscopy [143]. Indeed, in confocal fluorescence microscopes, both excitation and emission light coming from out-of-focus planes is largely blocked respectively by the illumination and the detector pinhole, avoiding image blurring and enhancing contrast with the dark background. Hence, images taken with CLSMs appear as thin optical sections, generating the so-called “optical sectioning” effect. The position of the planes in which the pinholes and the specimen are located is what relates such a microscopy technique to the term confocal, as they all are conjugate planes; consequently, the image of the illumination pinhole is in focus at both the specimen plane and the detector one. It is important to note that, differently from the previously described microscopy techniques, image acquisition in confocal microscopes happens in series, as only a single point in the object is illuminated, and a scan of the specimen is required to obtain a complete image [158]. Laser scanning characterizes CLSM, which is the most used method for confocal microscopy [143,158].

**Figure 14:**
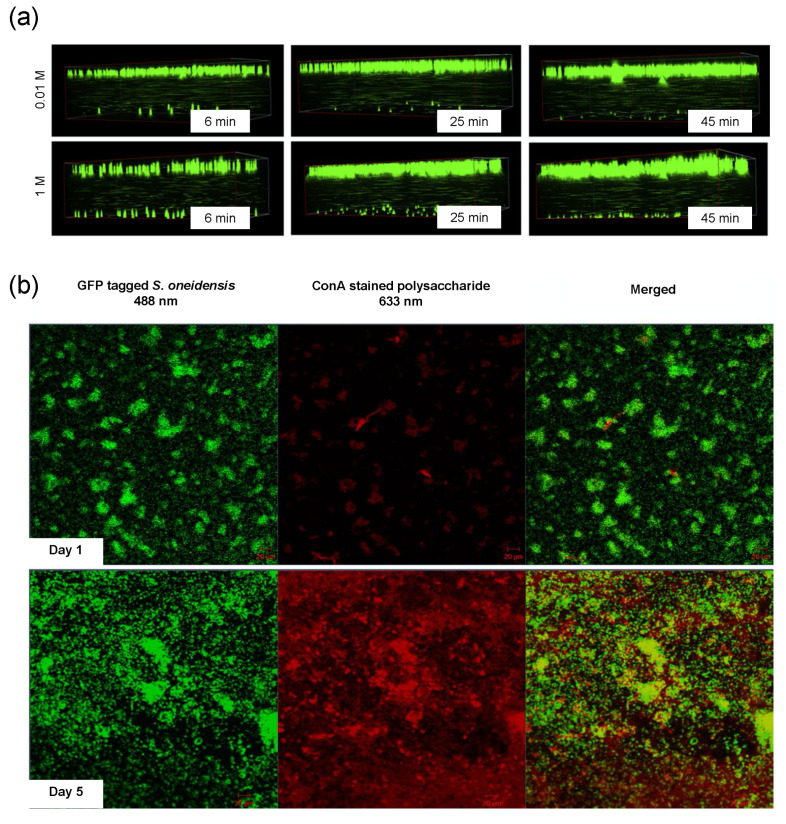
(**a**) Three-dimensional CLSM images of the microfluidic filtration device developed by Di et al. at different filtration times. Experiments were performed at two different KCl concentrations to account for the effects of ionic strength on the fouling process. Channel depth 25±2 μm. (**b**) CLSM images of multiple stained samples: GFP-tagged S. oneidensis cells (green) and ConA-stained extracellular polysaccharides (red) on a FO membrane after 1 and 5 days of processing. Adapted from [159,160].

Besides the higher resolution and contrast, CLSM has the advantage of enabling a 3D reconstruction of the specimen by combining images taken at different axial levels with the help of dedicated software. This represents a key aspect for the study of the fouling phenomena as, even observing the membrane from above, it is possible to reconstruct the fouling layers and analyze their structure. This feature was exploited by Di et al. for the dynamic visualization and quantification of latex particles deposition in a microfluidic filtration system specifically designed for CLSM coupling (Figure 14a) [159]. From 2D images, it was possible to observe that varying salt concentration in the particle suspension affected the initial stages of the deposition process with a higher presence of large aggregates at higher KCl concentrations. On the other side, 3D images clearly showed that particles formed a monolayer independently of the KCl concentration of the solution, suggesting that the growth of the aggregates happened parallel to the membrane surface rather than perpendicularly. In a subsequent study, the same microfluidic filtration setup was used for a detailed study on the dependance of particle fouling from pH, ionic strength and salt concentration in the feed. Once again, the combination of 2D and 3D images allowed to distinguish between different fouling dynamics, e.g., the preferential formation of aggregates at lower pH, that are directly correlated to particle–particle and particle–membrane interactions (Figure 14a). Together with fluorescence microscopy, CLSM can also take advantage of the use of different fluorophores to better investigate fouling dynamics in presence of more foulant species. This helped Marty et al., in their already mentioned work, to distinguish the presence of EPS all around the bacteria forming streamers and conclude that such structures were indeed formed by the contribution of both the foulants [125]. Multiple stains were also used by Mukherjee et al. to study the influence of EPS on biofouling in FO (Figure 14b) [160].

Specifically, the authors developed a CLSM-compatible membrane-embedded microfluidic flow cell to investigate biofouling nondestructively and showed that, despite the long-term biofilm dispersal, permeate flux did not increase due to the presence of EPS, which irreversibly blocked membrane pores.

Nevertheless, CLSM has some drawbacks too. First of all, the scanning process can represent a problem for dynamic observations. In fact, the first stages of the fouling phenomenon occur within very short times, which may not be sufficient for the microscope to acquire an image. Moreover, the tangential flux of fluorescent foulants over the membrane can result in blurred and noisy images, if the concentration is too high. The objectives’ working distance can be an issue as well, although microfluidic systems can usually be visualized with common commercial confocal objectives.

### 4.2. Optical Coherence Tomography

Optical coherence tomography (OCT) is an interferometric technique that enables 3D visualization of inhomogeneous samples, such as a fouled membrane, through the progressive acquisition of high-resolution cross-sectional images [161,162]. It is a non-invasive technology based on light interference where a near-infrared light beam is split to follow two different paths. The former travels axially through the sample and is partially reflected every time there is a change in the refractive index, e.g., in the presence of a cake layer on the membrane surface. The other beam crosses the so-called reference arm, which is equipped with a mirror and whose length is precisely determined. Due to reflections caused by the specimen’s heterogeneity, the sample beam travels a path of different length compared to the reference one, therefore generating a series of interferences when the two are combined. The associated variations in light intensity are measured by a detector and the resulting interferograms are processed to obtain a final 2D image. It is important to note that in order for interference to be observed, light has to be coherent, or rather the phase difference between the waves of two beams has to be constant. OCT makes use of broadband low-coherence light, for which phase differences remain constant only within a short length usually in the order of micrometers [162].

In its early development, the cross-sectional scan of the specimen was performed modulating the reference arm length for each depth by moving the reference mirror. In this mode, called time-domain OCT (TD-OCT), 2D images are acquired by scanning the sample point-by-point first alongside its depth (A-Scan) and then moving laterally across the width of its section (B-Scan). 3D images can be obtained by the composition of several B-Scans. TD-OCT has currently been replaced by Fourier domain OCT (FD-OCT), characterized by the absence of moving parts. In FD-OCT, the intensity signal is recorded as a function of frequency, rather than distance, and A-Scans are then computed via Fourier transformation; in doing so, a single scan provides information about the whole depth of the sample, resulting in a higher imaging speed [163]. Compared to CLSM, in which samples are optically sectioned too, OCT has a higher penetration depth owing to the long-wavelength light sources [164,165]. However its spatial resolution is very limited, and due to the absence of any stain, it is impossible to distinguish between different species, making such a technique not suitable to investigate the interplay of multiple foulants at once [163].

OCT is an already-established technique in the biomedical field, where it is used for tissue diagnosis when a biopsy is not applicable and represents a high-potential technique for in situ real-time monitoring of the fouling phenomena thanks to the possibility of monitoring foulant accumulation, distribution and morphology. FD-OCT application to biofouling monitoring is very common since such methodology allows one to investigate biofilm formation in situ over long times and without stressing the microorganisms. Park et al. used OCT to monitor biofouling in an RO filtration cell over 50 days, clearly observing the various stages characterizing such phenomena [166]. Specifically, 2D images showed no changes in the membrane surface for the first 11 days, during which the conditioning stage was taking place. Due to colonization and growth, a considerable increase in the thickness of the biofilm was instead observed from day 20 to 35, after which no increases were recorded, implying an equilibrium between the rate of growth and detachment. The authors also underline the importance of a 3D representation, as the use of single 2D cross-sectional images for biovolume quantification is highly affected by uncertainty.

Biofilm development was also investigated by Quian et al., who developed a tortuous microfluidic device to study the role of biofouling in irrigation devices, and by Weiss et al., who used Doppler optical coherence tomography (DOCT) to monitor local hydrodynamics in a single microfluidic channel [167,168]. DOCT exploits the Doppler effect—i.e., the frequency shift experienced by the waves reflected from a moving object—and allows quantitative imaging of fluid flow, thus giving supporting information to the structural ones provided by FD-OCT, without the need of additional independent measurements [161]. In such a way, it was possible to directly observe the dynamic deformation of the biofilm surface due to the shear stresses exerted by the fluid [168]. OCT and DOCT were also used by Gao et al. to study the formation of a cake layer of bentonite particles during a FO process and the influence of a membrane spacer on the fouling layer growth [169].

### 4.3. Nuclear Magnetic Resonance Imaging

Nuclear magnetic resonance (NMR) imaging—also known as magnetic resonance imaging (MRI)—is a non-invasive investigation technique that takes advantage of the intrinsic magnetic properties of protons in atomic nuclei and allows to access the membrane module inner structure even in cases of opaque or non-transparent units [170,171,172,173]. In an NMR experiment, the sample is firstly placed in a static magnetic field that triggers the magnetization of the specimen protons to align with or against the direction of the field, depending on the energy level. Indeed, due to their magnetic moment, atomic nuclei behave similarly to microscopic bar magnets, which, in the absence of any external magnetic field, are randomly oriented. The excitation step is thus realized by means of a second external oscillating magnetic field generated by radiofrequency coils; such a field is meant to provide an exact quantum of energy to induce an energy level transition in the protons, i.e., the spin flip. In the subsequent relaxation step, as soon as the oscillating field is shut off, protons spontaneously return in their equilibrium state, releasing the previously absorbed energy and thus emitting a signal, which can be recorded and translated to create spectra or images representing variations within the sample.

**Figure 15:**
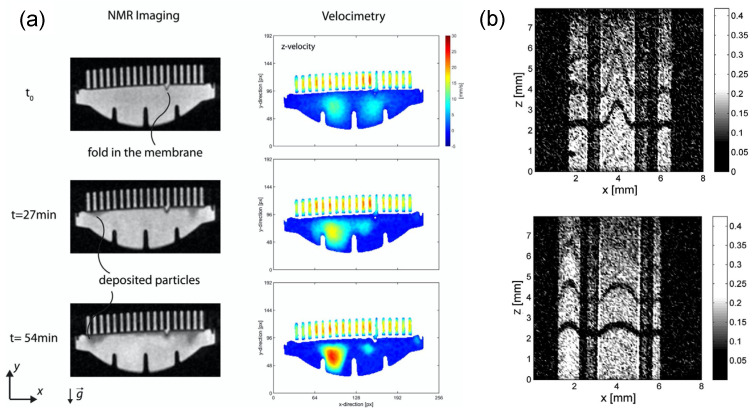
(**a**) MRI and flow-MRI of the commercial filtration module investigated by Wiese et al. NMR imaging clearly shows the presence of a cake layer after 54 min of filtration (darker areas). (**b**) MRI saturation stripes indicating axial velocity inside and outside a single hollow fiber membrane. Images show the effects of the absence (top) and presence (bottom) of ${\mathrm{Ca}}^{2+}$ ions on fouling development due to alginate deposition after 22 and 21 min [175,181].

One of the most important concepts on which NMR imaging relies is nuclear shielding [170]. Atoms in the specimen are surrounded by electron clouds, which orbit the nuclei influencing the magnetic field experienced by the latter; changes in the chemical environment of the atom affects the energy level of the nucleus resulting in different radio frequency required during the excitation step. Owing to nuclear shielding, NMR not only enables a structural characterization of the sample, but also a chemical one. Moreover, through the use of gradient coils, which linearly vary the applied magnetic field, NMR can also provide information regarding the local position and velocity in flowing samples, such as feed and permeate currents in a membrane module [174]. As showed by Wiese et al., this information is of crucial importance especially for the design of efficient membrane filtration modules [175]. With the help of MRI, the authors investigated the flow field and the development of a fouling layer made of colloidal silica in a commercial sterile membrane filter (Figure 15a). Quantifying the local flux distribution and visualizing particle deposition over time, it was possible to correlate the evolution of fouling to the feed flow and evaluate the active membrane area inside the module, thus showing all the critical design aspects affecting filtration performances.

Despite the latest technological advances regarding NMR systems, the application of this technique to microfluidics still represents a challenge. Conventional MRI spatial resolution is indeed limited by an inherent low signal-to-noise ratio, which is further aggravated by the small fluid volumes typical of microfluidic devices. This leads to very low-intensity signals hardly detectable even with high-field magnets and optimized radio frequency circuits [176]. Several works have focused on the implementation of novel approaches aimed at improving MRI resolution for microfluidic applications. McDonnel et al. and Paulsen et al. enhanced MRI sensitivity using remote detection, that is, physically separating the signal detection from the other steps of the experiment [177,178,179]; a nominal isotropic spatial resolution of 2.8 μm was instead obtained for the first time by Chen and Tycko, who exploited low temperatures (28 K) where NMR signals are boosted by factors such as lower thermal noise [176].

NMR imaging was demonstrated to be a powerful technique to study the fouling phenomena in hollow fiber membranes, commercially available with diameters down to 100 μm [92]. Via NMR imaging, Çulfaz et al. investigated the influence of particle dimension on the evolution of fouling in a single hollow fiber, observing that while larger particles formed a highly concentrated cake layer on the membrane surface, smaller ones produced a thicker yet less-concentrated CP layer, resulting in lower resistance against permeate flux, compared to the previous case [180]. In the work of Arndt et al., MRI enabled the in situ observation of fouling development due to alginate deposition and the influence of ${\mathrm{Ca}}^{2+}$ on this process [181]. Namely, in the absence of ions, it was possible to observe an unstructured CP layer, which resulted in lower NRM intensity areas. MRI also allowed to study the effect of fouling on the flow distribution inside the feed channel of the membrane, obtaining 2D, spatially resolved velocity profiles at the center of the membrane lumen by saturation stripes (Figure 15b). Due to the diameter, the hollow fiber membranes examined in these studies can be more appropriately classified as millifluidic, rather than microfluidic; however, they represent an important step for the implementation of NMR imaging to investigate the fouling phenomena in microfluidic systems.

### 4.4. Other Emerging Techniques

In the previously described approaches, microfluidics represented an essential tool to study fouling at the microscale. Nevertheless, there are many other techniques suitable for the dynamic investigation of such phenomena, which have been implemented for microfluidic applications and are currently adopted on a macroscopic level, yet scarce or no application can be found regarding their use to study fouling in microfluidic systems.

#### 4.4.1. Raman Spectroscopy

Raman spectroscopy (RS) is a vibrational spectroscopy method that allows one to obtain the chemical fingerprint of material by assessing vibrations and rotations of the functional groups of its molecules. Such a technique can thus be used to characterize different species with a high detection sensitivity, making it especially suitable for the investigation of the early stages of fouling [182,183,184]. The key principle of RS is the Raman shift, that is, the change in wavelength of a photon scattered by a molecule in the sample. Indeed, in RS, the specimen is shined with a laser of a definite wavelength, whose light will mostly be scattered without any wavelength change, i.e., elastically (Rayleigh scattering); only 1 in $10^{5}$–$10^{7}$ photons will be subject to inelastic scattering (Raman scattering), experiencing the Raman shift characteristic of the molecules the light interacts with [185].

The reduced number of photons undergoing inelastic scattering constitutes the main drawback of Raman spectroscopy; in fact, the weak intensity of the Raman scattering makes it difficult to distinguish the Raman peaks from the background noise generated by the environment surrounding the sample. Signal intensity can be enhanced by modifying the surface of interest with metallic nanoparticles as in surface-enhanced Raman spectroscopy (SERS) [186], the most common technique used to study membrane fouling [141]. Thanks to the confined environment provided by chips and capillaries, microfluidics represents a high-potential tool to overcome the main limitations of SERS applied to membrane filtration, namely the probability of detachment of nanoparticles due to high cross-flow velocity and the disturbances generated by high pressures and vibration in a macroscopic membrane module [187]. The immobilization of nanoparticles, however, can also affect membrane performances as particles might act as foulants, blocking or narrowing pores.

To the best of our knowledge, there are not any studies concerning the application of RS to study fouling in microfluidic devices; however, some researchers have recently exploited the advantages of membrane separation to further improve molecular detection via RS. A microfluidic implementation of SERS was proposed by Chang et al., who developed a detection device equipped with a membrane whose purpose was to concentrate a bacterial suspension and separate metabolites for the subsequent SERS aimed to the determination of antibiotic susceptibility [188]. In another study, Krafft et al. set up a microfluidic chip equipped with a membrane, which fulfilled two functions at the same time, namely enriching the liquid sample and hosting the nanoparticles necessary for the SERS detection [189].

#### 4.4.2. Fourier Transform Infrared Spectroscopy

Fourier transform infrared (FT-IR) spectroscopy is an absorption-based spectroscopy technique in which a sample is exposed to infrared radiation (IR), whose absorption results in an increase of the vibrational and rotational energy of the sample molecules [190]. FT-IR spectroscopy makes use of an interferometer to scan all the frequencies in the IR region generated by the source and Fourier transform is used to convert the interferogram from the time domain to the frequency one. Each species converts the absorbed radiation in a unique way, thus FT-IR spectroscopy is able to identify and characterize the composition of a material with high specificity [191].

Compared to other investigation techniques such as fluorescence microscopy, FT-IR spectroscopy does not need any labeling agent, which makes it suitable especially in the study of biofouling, as the addition of a fluorophore might potentially affect system physiology [192]. In addition, differently from RS, FT-IR spectroscopy is characterized by higher signal yields relative to the incident power. The application of FT-IR spectroscopy to real-time monitoring of fouling in filtration devices is nonetheless limited by the interference arising from the broad O-H vibration bands of water. A strategy to overcome such a problem can be the use of microfluidics [193]. Indeed, the low volumes processed in microfluidic chips allow one to obtain a very thin layer of water between the sample and the path of the incident radiation, thus containing the detrimental effects of water absorption. Holman et al. combined FT-IR spectroscopy with open channel microfluidics to study biofilm growth in different geometries and monitor the biochemical response of the system over time [194]. With such a system, it was possible to correlate the growth dynamics to the presence of the principal components of the EPS.

In order to be used for FT-IR spectroscopy, microfluidic devices have to be made of IR transparent materials, which can be expensive and hard to craft compared to glass and polymeric materials such as PDMS or polycarbonate. Chan et al. proposed a versatile approach for the prototyping of reusable microfluidic devices compatible with FT-IR spectroscopy [195]. Device manufacturing consisted of the printing of wax on the surface of an IR transparent substrate, followed by the positioning of a second layer to generate closed channels of desired thickness. This methodology was subsequently exploited for live imaging of single cells [196].

#### 4.4.3. Ellipsometry

Ellipsometry is a spectroscopy technique based on light polarization commonly employed to characterize materials surfaces. In an ellipsometry measurement, a monochromatic light beam illuminates the surface of the specimen with equal parallel and perpendicular polarization components; due to the interaction with the surface, reflected light is elliptically polarized. The signal detected from a photodetector is subsequently processed and compared to a specific mathematical model, whose selection depends on the expected numbers of layers within the sample itself. This constitutes a limiting factor especially when dealing with complex and heterogeneous materials such as fouled membranes [197]. Among the information one could gather using ellipsometry (e.g., surface roughness, crystalline nature, electrical conductivity, refractive index), this optical technique is particularly effective in the real-time investigation of adsorption processes on various surfaces and its most popular application involves the thickness measurements of films and layers, with a definition down to the nanometer scale [197,198].

The number of studies involving ellipsometry to investigate the fouling phenomena in microfluidics is limited. However, the coupling of this technique and confined environments could have a positive impact especially on the study of the early stages of fouling. Indeed, these phases are characterized by adsorption reactions at the liquid–solid interface between the feed and the membrane, whose investigation requires small volumes in order to minimize light dispersion [199,200]. Huber et al. exploited ellipsometry to investigate the performances of a polymeric coating capable of adsorbing or releasing proteins as a function of temperature [201]. Although adsorption/release tests were performed in a microfluidic chip equipped with several micro heaters, the thickness of the protein layer was only measured ex situ. Another application of ellipsometry for antifouling coatings can be found in the work of Peterson et al., who studied PDMS biocompatibility, proving how the adsorption of proteins such as fibrinogen can hinder coating antifouling effects, allowing the proliferation of cell colonies during incubation experiments [202]. The design of a microfluidic optical cell for in situ ellipsometry was proposed by Kondoh et al., who significantly reduced the cell volume, compared to typical trapezoidal flow cells, by using a commercial glass slide functioning as a cover for the fluid space and a transparent window for measurements [199].

#### 4.4.4. X-ray Microimaging

X-ray microimaging (XMI) is a non-invasive in situ imaging technique, which relies on the penetration of high-energy electromagnetic radiation into a material, whose refractive index influences both the amplitude and the phase of the x-rays [203]. Aided by synchrotron radiation, XMI can reach spatial resolutions up to 1 μm, thus having the potential to provide quantitative data in terms of pore blocking, cake thickness and also membrane morphology, since it allows to investigate membrane’s inner structure. In addition, 3D reconstructions of a sample can be performed [204].

**Figure 16:**
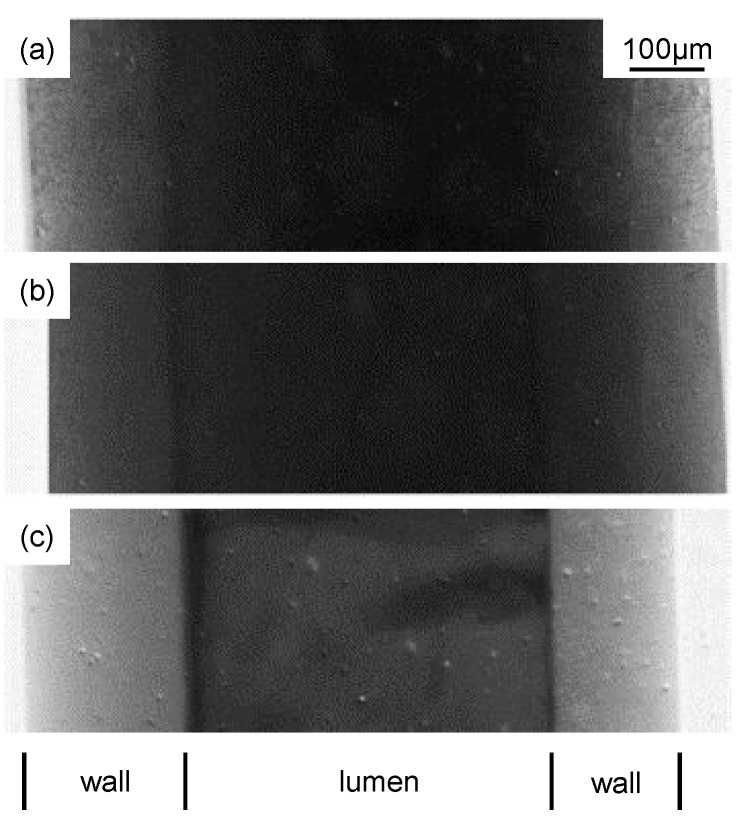
XMI of a membrane fiber during progressive deposition of ferric hydroxide on the lumen wall after (**a**) 0 min, (**b**) 5 min and (**c**) 30 min of operation. The absorptive contrast on the wall gradually increases as the deposition becomes thicker. Adapted from [205].

The first application of XMI to membrane processes observation was proposed by Yeo et al. [205]. Using such an imaging technique, the authors were able to study iron hydroxide particles deposition both inside the pores and on the lumen surface of a single hollow fiber of 600 μm inner diameter (Figure 16). In addition, images evidenced the presence of air bubbles, proving the possible application of XMI to the identification of two phases phenomena [206]. In a recent study, XMI was applied to directly visualize water droplet spreading and penetration inside a membrane in an oily environment [207].

Despite being a powerful investigation technique for fouling investigation, the application of XMI to microfluidic systems is mainly related to the field of flow measurements and velocimetry [208,209,210].

## 5. Perspectives

Due to the advantages offered by the characteristic micro-confined environments, the use of microfluidics for the investigation of fouling represents a high-potential strategy for the comprehension of all the complex phenomena taking place during filtration processes. In this review, the various stages characterizing the evolution of fouling were discussed, enlightening the developments enabled by the adoption of a microfluidic approach. The interpretation of fouling via a mathematical approach was then investigated, and various models, based on flux and pressure data, were examined assessing their principal advantages and drawbacks. Lastly, several dynamic in situ investigation techniques, in which microfluidics plays a key role, were reviewed. Such methodologies represent a possible solution to the limitations related to the study of fouling by means of measuring permeate fluxes or TMP variations, as they allow direct visualization of all the phenomena taking place at the microscale with unprecedented time and spatial resolutions.

Fouling has always represented a major technical challenge for microreactors and membrane processes and it still gains attention from both researchers and industries, particularly interested in the newest trends involving fouling mitigation through membrane modification and the development of innovative approaches aimed to directly monitor the evolution of the process. As shown in this review, the implementation of microfluidics constitutes a great scientific opportunity for the elucidation of the still-debated aspects of fouling phenomena. Further improvements are expected, powered by the latest technological advancements in terms of materials and fabrication techniques for microfluidics, with many new applications of such tools to already-established investigation methodologies to be seen in the near future.

Forthcoming developments in microfluidic technologies will also be crucial for the growth of nanofluidics, a branch that studies fluid transport in or around objects whose characteristic dimensions can go below 100 nm. Indeed, in this context, microfluidics constitutes a joining link between the macroscopic world and the nano-scale [211]. Owing to the extremely high molecular selectivity, nanofluidics represents an interesting tool, especially for water desalinization and purification processes; however, the drawbacks of fouling are much more detrimental here than in microfluidic environments [212]. Indeed, nanofluidic devices are widely adopted for applications such as single-molecule handling and detection, yet the processing of mixtures containing different foulants can be troublesome [213]. Nanofluidic systems constitute a high-potential technology for the investigation of the fouling dynamics taking place below the microscale, like those involving the presence of an electrical double layer or molecular-scale interactions arising in the early stages of the process.

## Figures and Tables

**Table 1:** Membrane fouling models for constant-pressure dead-end and crossflow filtration.

Filtration Model	n	Dead End [129,131]	Crossflow [126,135]
Complete blocking	2	$J\left(t\right)=J_{0}\;\exp\left(-K_{CPB}t\right)$	$J\left(t\right)=J^{*}+\left(J_{0}-J^{*}\right)\;e^{-J_{0}K_{CPB}t}$
Standard blocking	3/2	$J\left(t\right)=\frac{J_{0}}{{\left(K_{SPB}\;J_{0}{}^{1/2}t+1\right)}^{2}}\;$	$J\left(t\right)=\frac{J_{0}}{{\left(K_{SPB}\;J_{0}{}^{1/2}t+1\right)}^{2}}$
Intermediate blocking	1	$J\left(t\right)=\frac{J_{0}}{\left(K_{IPB}\;J_{0}t+1\right)}$	$J\left(t\right)=\frac{J_{0}\;J^{*}\;e^{K_{IPB}\;J^{*}t}}{J^{*}+J_{0}\left(e^{K_{IPB}\;J^{*}t}-1\right)}$
Cake filtration	0	$J\left(t\right)=\frac{J_{0}}{{\left(1+2K_{CLF}\;J_{0}{}^{2}t\right)}^{1/2}}$	$t=\frac{1}{K_{CLF}\;J^{*}{}^{2}}\ln\left[\left(\frac{J\left(t\right)}{J_{0}}\;\frac{J_{0}-J^{*}}{J\left(t\right)-J^{*}}\right)-J^{*}\left(\frac{1}{J\left(t\right)}-\frac{1}{J_{0}}\right)\right]$

**Table 2:** Bolton’s combined models in terms of V=f(t) for constant pressure filtration.

Filtration Model	Component Mechanisms	Equation
Cake—complete	Cake filtration, complete blocking	$V\left(t\right)=\frac{J_{0}}{K_{CPB}}\left(1-\exp\left(\frac{-K_{CPB}}{K_{CLF}J_{0}{}^{2}}\left(\sqrt{1+2K_{CLF}J_{0}{}^{2}t}-1\right)\right)\;\right)$
Cake—intermediate	Cake filtration, intermediate blocking	$V\left(t\right)=\frac{1}{K_{IPB}}ln\left(1+\frac{K_{IPB}}{K_{CLF}J_{0}}\left({\left(1+2K_{CLF}J_{0}{}^{2}t\right)}^{\frac{1}{2}}-1\right)\;\right)$
Complete—standard	Complete blocking, standard blocking	$V\left(t\right)=\frac{J_{0}}{K_{CPB}}\left(1-\exp\left(\frac{-2K_{CPB}t}{2+K_{SPB}J_{0}t}\right)\right)$
Intermediate—standard	Intermediate blocking, standard blocking	$V\left(t\right)=\frac{1}{K_{IPB}}ln\left(1+\frac{2K_{IPB}J_{0}t}{2+K_{SPB}J_{0}t}\right)$
Cake—standard	Cake filtration, standard blocking	$V\left(t\right)=\frac{2}{K_{SPB}}\left(\beta \cos\left(\frac{2\pi }{3}-\frac{1}{3}\arccos\left(\alpha \right)\right)+\frac{1}{3}\right)$, $\alpha =\frac{8}{27\beta^{3}}+\frac{4K_{SPB}}{3\beta^{3}K_{CLF}J_{0}}-\frac{4K_{SPB}{}^{2}t}{3\beta^{3}K_{CLF}}$, $\beta =\sqrt{\frac{4}{9}+\frac{4K_{SPB}}{3K_{CLF}J_{0}}+\frac{2K_{SPB}{}^{2}t}{3K_{CLF}}}$
